# Sorption of Oxybenzone onto Polystyrene Microplastics Influences Bioavailability and Early-Life Development in Zebrafish *(Danio rerio*)

**DOI:** 10.3390/toxics14030239

**Published:** 2026-03-10

**Authors:** Melissa I. Ortiz-Román, Marielisa Soto-Parrilla, Karla I. Capó-Romero, Adriana S. Torres-Rodríguez, Félix R. Román-Velázquez

**Affiliations:** 1 Department of Chemistry, University of Puerto Rico, Mayagüez Campus, Mayagüez, PR 00681, USA; marielisa.soto@upr.edu (M.S.-P.); felixr.roman@upr.edu (F.R.R.-V.); 2 Pre Med Program, Department of Biology, University of Puerto Rico, Mayagüez Campus, Mayagüez, PR 00681, USA; karla.capo@upr.edu; 3 Industrial Biotechnology Program, Department of Biology, University of Puerto Rico, Mayagüez Campus, Mayagüez, PR 00681, USA; adriana.torres37@upr.edu

**Keywords:** oxybenzone, polystyrene microplastics, zebrafish, embryos, larvae, toxicology, adsorption capacity

## Abstract

Oxybenzone (BP-3) and polystyrene microplastics (PS MPs) are pervasive aquatic contaminants whose combined biological effects remain insufficiently characterized. This study investigated co-exposure to BP-3 and PS MPs in zebrafish embryos (*Danio rerio*), focusing on developmental endpoints, tissue bioaccumulation, and time-dependent sorption behavior. Embryos were exposed to 0.10–1.50 mg/L BP-3 for 96 h in the presence of PS MPs. Mortality, developmental abnormalities, and tissue BP-3 concentrations were measured, and chemical analysis was performed by HPLC-DAD. Although mortality was not significantly affected, embryos exhibited developmental abnormalities, particularly in swim bladder formation. Tissue BP-3 accumulation increased with exposure concentration. The influence of PS MPs on BP-3 uptake was concentration-dependent: at lower BP-3 exposures, PS MPs reduced tissue accumulation, whereas at higher exposures this reduction became negligible or was no longer observed. This suggests a dual role for PS MPs: mitigating direct aqueous exposure by sequestering BP-3 yet simultaneously acting as potential vectors for its environmental persistence and trophic transfer through alternative pathways. Independent time-resolved experiments showed rapid BP-3 removal from the aqueous phase in the presence of PS MPs, with early stabilization consistent with rapid partitioning behavior. These findings highlight the complex interactions between emerging contaminants and MPs, underscoring the need for further research into their ecological implications.

## 1. Introduction

Marine plastic pollution has intensified in recent decades, with estimates indicating that between 15 and 51 trillion plastic fragments, predominantly microplastics (MPs) (1 µm–5 mm), are currently present in the oceans [1,2]. MPs are not only physical waste but also emerging environmental contaminants that can chemically interact with other compounds in aquatic environments [3]. Through these interactions, MPs can act as vectors for contaminant transport, increasing the exposure of aquatic organisms to potentially toxic substances [4,5,6,7]. Numerous studies have reported adverse biological effects associated with exposure to MPs and related contaminants, including developmental abnormalities, bioaccumulation, oxidative stress, cellular damage, endocrine disruption, and mortality [8,9,10,11]. However, their ability to adsorb chemicals may also temporarily reduce the concentration of freely dissolved organic compounds in water [3,4,8,9,10,11,12], thereby decreasing their bioavailability. This effect is particularly relevant for organisms whose exposure is driven primarily by direct uptake through the gills or other thin epithelial tissues, especially during short-term exposures and in the absence of ingestion [9,10,11,12,13].

Among the wide range of organic contaminants detected in aquatic environments, BP-3 has emerged as a compound of particular concern due to its widespread use as a UV filter in cosmetic products, its environmental persistence, and its bioaccumulative potential [14,15]. BP-3 has been detected in several aquatic species, including zebrafish, brine shrimp, and clams, where adverse biological effects have been documented [16]. Previous research has demonstrated that BP-3 induces developmental abnormalities and biochemical alterations in zebrafish embryos, as well as intestinal damage and increased mortality in brine shrimp, underscoring its toxicity during early life stages [8,10,11,12]. Additionally, exposure to BP-3 has been associated with oxidative stress, lipid peroxidation, neurotoxicity, and tissue-specific damage in marine invertebrates such as the clam *Scrobicularia plana* [10]. In this context, although the typical background concentration of BP-3 in natural waters is generally reported in the low µg/L range (~1 µg/L), higher levels have been documented in localized environments, including coastal and recreational waters, reaching up to 1.395 mg/L [14]. The concentrations used in this study (0.10–1.50 mg/L) were selected to represent high-exposure scenarios and ranges previously shown to induce biological effects in zebrafish [17]. These findings highlight the importance of understanding the environmental factors that modulate the distribution, persistence, and bioavailability of BP-3 in aquatic ecosystems.

Polystyrene (PS) is among the most prevalent MPs detected in aquatic environments due to its extensive production and widespread use in packaging, insulation, and food-related materials [11,18]. With a density of approximately 1.04–1.06 g/cm^3^, typically higher than that of polyethylene (PE) MPs, PS may be distributed across both surface waters and sediments [11,19]. Its sorption behavior is strongly influenced by its chemical structure; specifically, the presence of aromatic rings enables π–π interactions with aromatic organic contaminants, resulting in greater sorption relative to non-aromatic polymers such as PE and polyvinyl chloride (PVC) [12]. Experimental microplastic studies commonly employ particle loads that guarantee effective exposure and allow evaluation of vector effects under controlled laboratory conditions [20]. Due to the relatively large particle size (900 µm) and the known tendency of microplastics to sediment in aqueous media, a mass of 25 mg PS was selected to ensure sufficient particle availability and surface area for interaction with BP-3. Given the strong sorption potential of PS and the known toxicity of BP-3, it is essential to investigate how their interaction may influence biological responses in aquatic species. In this study, zebrafish embryos were selected as the biological model due to their rapid development, body transparency, and established use in the OECD TG 236 Fish Embryo Toxicity (FET) test, which allows sensitive detection of acute and sublethal toxic effects [21,22,23].

Despite the well-documented individual toxicities of BP-3 and the substantial sorption capacity of PS, the interactive effects of their co-exposure on aquatic organisms, particularly how PS may modulate BP-3 bioavailability and toxicity, remain poorly understood. To address this critical knowledge gap, the present study examines how PS MPs modulate BP-3 toxicity and bioaccumulation in zebrafish embryos, providing insights into the environmental fate and interactive effects of these prevalent aquatic contaminants. Such understanding is essential, as the combined mechanisms and toxicological outcomes of co-exposure during early life stages of aquatic organisms remain insufficiently explored despite the widespread co-occurrence of these contaminants in natural environments.

## 2. Materials and Methods

### 2.1. Fish Maintenance and Breeding: Zebrafish

Adult zebrafish *(Danio rerio*) were obtained from Caribe Fisheries, Inc. (fish farm, Lajas, PR, USA) and housed in a ZebTEC Benchtop zebrafish housing system (Tecniplast, Buguggiate, VA, Italy) with mechanical filtration and sterilization (ultraviolet 40 W disinfection) under an automated 12/12 h light/dark cycle. The water temperature was set to 27 ± 1 °C, and the room temperature was 24 ± 1 °C. Reverse Osmosis (RO) + UV water, commercial sea salts (Instant Ocean^®^ Sea Salt, St. Blacksburg, VA, USA), and NaHCO_3_ (Sigma-Aldrich, St. Louis, MO, USA) were added to obtain pH and conductivity values of 7.0 ± 0.5 and 1000 ± 100 μS, respectively. This water with added substances is known as the system water. Properties such as pH, conductivity, system, room temperature, and system volume were checked and recorded daily. Adult fish were fed twice daily with Zeigler^®^ Adult Zebrafish Diet (Zeigler Bros, Gardners, PA, USA). The animal protocol was evaluated and approved by the Institutional Animal Care and Use Committee (IACUC) of the University of Puerto Rico at Mayagüez (Office of Laboratory Welfare Assurance number D20-01098).

### 2.2. Reproduction Process

**Scheme 1:**
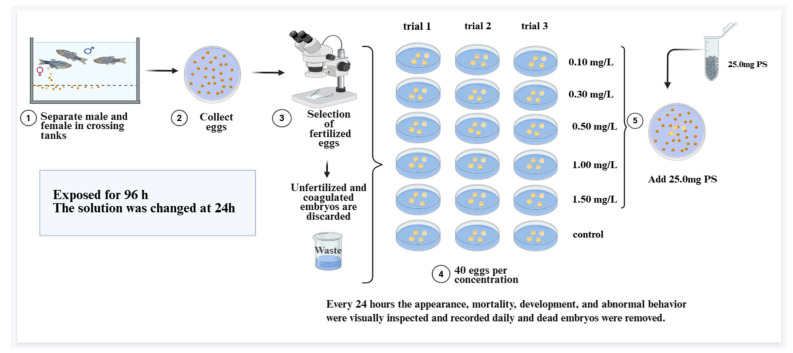
Scheme of the zebrafish embryo toxicity test procedure. Modified image [8] with permission. Created in BioRender (accessed on 19 February 2026). The experimental settings for egg production and collection, pre-exposure immediately after fertilization in glass Petri dishes, selection of fertilized eggs with a stereomicroscope, and distribution of fertilized eggs into Petri dishes prepared with their respective test concentrations were as follows.

The fish of interest were selected and placed in crossing tanks (approximately 50–75% filled with system water). The female-to-male ratio did not exceed 1:2 (Scheme 1). Adults *Danio rerio* used for egg production were 8–10 months old, corresponding to reproductively mature individuals. The crossing tanks were placed on a rack overnight under the same environmental conditions. The next morning, a 250 W light was turned on in the tanks to provide warmth and induce spawning. At noon, the light was turned off, and the embryos were collected.

### 2.3. Preparation of Xenobiotic (BP-3 + PS)

Stock solution of concentrated (200.0 mg/L) BP-3 (CAS: 131-57-7; Sigma-Aldrich, St. Louis, MO, USA) with HPLC-grade methanol (CAS: 67-56-1; Sigma Aldrich, St. Louis, MO, USA). The mixture was then placed in an ultrasonic bath (Bransonic^®^, model 3510R-MT, Danbury, CT, USA) for 15 min. The stock solution was then diluted with system water to prepare treatment concentrations of 0.10, 0.30, 0.50, 1.00, and 1.50 mg/L. The samples were then subjected to ultrasound for 30 min. These concentrations were selected based on environmental concentrations reported in other studies and their low water solubility [24]. The diluent was system water to simulate the natural environment of zebrafish embryos as closely as possible and avoid other variables that could affect them. For each treatment, 25 mg of PS Powder 900 µm (GoodFellow Cambridge Ltd., Huntingdon, Cambridgeshire, UK, 900 micron) were weighed, placed into plastic vials, and added to Petri dishes containing the BP-3 solutions at the specified concentrations.

### 2.4. Fish Embryo Acute Toxicity Test

Fertilized eggs were collected and washed with system water. Eggs were examined for vitality using a stereomicroscope (AmScope SM-2TZ-LED; Irvine, CA, USA) and randomly distributed to the experimental groups. The experimental design strictly followed OECD guideline 236 for fish embryo acute toxicity testing, consisting of six groups: a control group (system water) and five experimental groups (0.10, 0.30, 0.50, 1.00, and 1.50 mg/L of BP-3). Embryos were transferred to glass Petri dishes containing 15 mL of BP-3 treatment solution and 25.0 mg PS. This experiment evaluated the co-exposure of zebrafish embryos to BP3 and PS microplastics. For comparative purposes, the findings were contrasted with data from a previous experiment conducted in the same laboratory under identical conditions but without PS MPs [8]. Forty embryos were placed in each dish, and three replicates were evaluated per treatment. Embryos were continuously exposed for 96 h post-fertilization (hpf) at 28 ± 1 °C (Benchmark’s MyTemp^TM^ MiniDigital Incubators, H2200-H; Sayreville, NJ, USA) in glass Petri dishes under a 12:12 h light/dark photoperiod and were not fed during exposure. Solutions were changed every 24 h. Their appearance, mortality, development, and abnormal behavior were visually inspected daily. Embryos were assessed for the presence or absence of morphological abnormalities at the treatment-group level, rather than by quantifying the frequency of each abnormality type. The embryos were then recorded using a stereomicroscope, and dead embryos were removed. Images of random larvae were captured using a Trinocular Compound Microscope (OMAX M837SL; Kent, WA, USA) with an 18.0 MP Digital camera (OMAX A35180U3, USA). After completion of the experiment, the embryos were euthanized by placing them in an ice bath (0 °C) for 30 min.

### 2.5. Ultrasound-Assisted Emulsified Liquid–Liquid Microextraction (UA–ELPME) Followed by Liquid Chromatography with DAD

At 24 h intervals, 5 mL samples of each exposure treatment were simultaneously collected before changing the exposure solution for analysis. Then, 1.0 mL of hexane mixed isomers (98+%, CAS:92112-69-1, Alfa Aesar, Ward Hill, MA, USA) was added to the collected 5.0 mL of sample in a 15 mL glass centrifuge tube. The tube was vortexed for 10 s. The resulting solution was sonicated for 30 min and then centrifuged at 700 rpm for 5 min. The organic phase (100 µL) was transferred into a 1.5 mL HPLC vial. The extract was allowed to evaporate under a fume hood, and the residue was re-dissolved in 500 µL of HPLC-grade MeOH and filtered through a 0.45 µm syringe filter. All reagents and samples were previously filtered through a 0.45 µm syringe filter.

### 2.6. Tissue Extraction

**Scheme 2:**
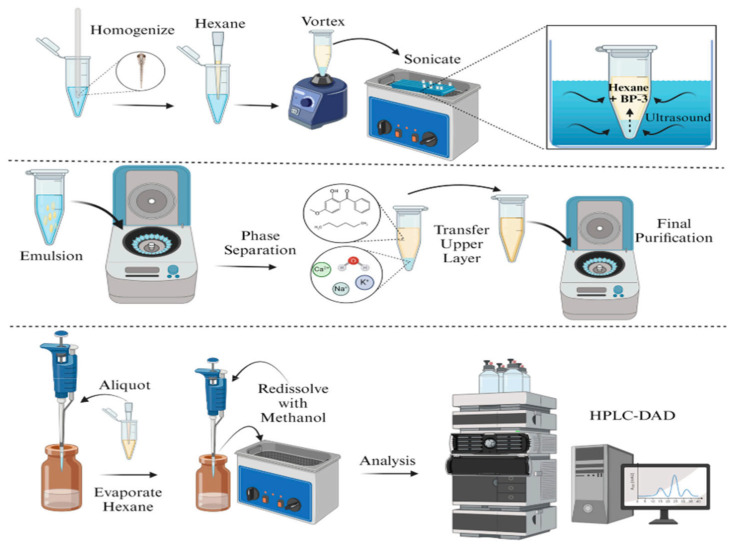
Ultrasound-assisted emulsified liquid-phase microextraction. Reproduced from [8], created with BioRender.

UA-ELPME was used to analyze zebrafish larval tissues (Scheme 2). The larval tissue was homogenized in a 1.5 mL centrifuge tube with 300 μL of deionized water. The extraction process involved adding 500 μL of hexane to the homogenized sample in a 1.5 mL centrifuge tube, then sonicating for 2 h. The sample was then centrifuged at 8000 rpm for 5 min, and the supernatant was transferred to a new centrifuge tube. For final purification, the sample was centrifuged at 8000 rpm, room temperature, for 5 min. Subsequently, 100 μL of the supernatant was transferred to an HPLC vial, evaporated, and then redissolved in 500 μL of HPLC-grade methanol.

### 2.7. BP-3–PS MP Exposure

Each test solution (100 mL), prepared as described in Section 2.3, was transferred into 100 mL amber glass bottles containing 500 mg of PS MPs (particle size: 900 µm). A control group, consisting of system water and 500 mg of PS MPs, was prepared in parallel for comparison. All bottles were placed on a horizontal shaker and agitated at 100 rpm for 48 h to allow interaction between BP-3 and the PS MPs.

Aliquots (5 mL) were collected from each bottle at 0, 1, 2, 4, 8, 24, and 48 h to assess the time-dependent behavior of BP-3 in the presence of PS MPs. Sample extraction and preparation for chromatographic analysis were performed as described in Section 2.5.

### 2.8. Quantification of BP-3

Sample analysis was conducted using a High-Performance Liquid Chromatography (HPLC) system (Agilent 1200 Series) equipped with an automatic injector and diode array detector (DAD). The HPLC conditions were as follows: separation was performed on a C18 column (Phenomenex, Madrid Avenue, Torrance, CA, USA) with dimensions of 150 × 4.60 mm and a 5 µm particle size. The mobile phase, comprising HPLC-grade methanol and deionized water in a 90:10 ratio, was continuously vacuum-degassed. The column was maintained at 25 °C with a flow rate of 1.0 mL/min for chromatography. The detection was performed at 289 nm. The Agilent ChemStation software (Rev. B.04.02 (96)) managed the LC 3D systems (Agilent Technologies, 2001–2009; Santa Clara, CA, USA).

### 2.9. Statistical Analysis

All statistical analyses were performed using Minitab Statistical Software (version 21.1 (64-bit)). Data are expressed as the mean ± standard deviation (SD). Differences were determined by one-way analysis of variance (ANOVA), followed by appropriate post hoc tests, such as Dunnett’s test for comparisons against control, Tukey’s HSD for all pairwise comparisons, and Fisher LSD method, where significant differences (*p* < 0.05) were detected.

## 3. Results and Discussion

### 3.1. Visual Inspection

**Figure 1:**
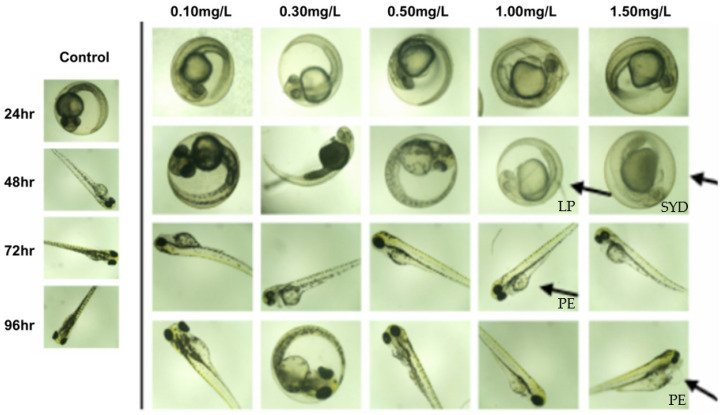
Pictures were taken using a Trinocular Compound Microscope (OMAX M837SL; Kent, WA, USA) with an 18.0 MP Digital camera (OMAX A35180U3) every 24 h for 96 h of treatment of exposed zebrafish larvae with 0.00, 0.10, 0.30, 0.50, 1.0, and 1.5 mg/L of BP-3. Deformities and reduced pigmentation were observed in embryos at higher BP-3 concentrations. (*n* = 3).

Morphological abnormalities were observed in all BP-3 exposure groups, whereas no such alterations occurred in the control embryos. The highest concentrations (1.00 and 1.50 mg/L) induced visible malformations within the first 24 h post-fertilization. The most frequently observed abnormalities included severe yolk deformation (SYD), lack of pigmentation (LP), and pericardial edema (PE), with PE showing the greatest prevalence at 1.00 and 1.50 mg/L after 72 h of exposure (Figure 1).

Two primary mechanisms reported in the literature may explain these developmental defects. One is oxidative stress triggered by BP-3. Downs (2016) demonstrated that BP-3 induces oxidative stress in coral tissues [20], and Zhang similarly showed that ultraviolet light can exacerbate oxidative stress caused by benzophenone-type UV filters in fish [18]. Oxidative stress arises when reactive oxygen species (ROS) production surpasses antioxidant capacity, leading to lipid peroxidation, protein carbonylation, and DNA damage [25]. Because balanced ROS levels are essential for normal embryogenesis, excessive ROS generation can disrupt developmental processes and has been associated with morphological defects such as edema and impaired pigmentation [24].

Due to consistently low mortality across treatments, reliable estimates were not possible for calculating the LC_50_.

**Figure 2:**
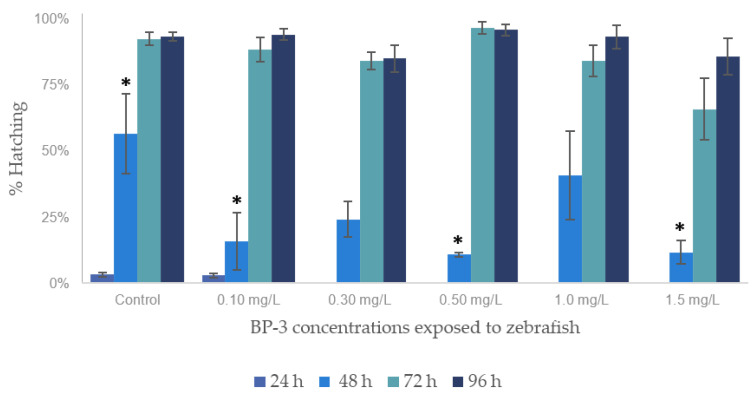
Percentage of zebrafish embryo hatching following exposure to BP-3 (0.00, 0.10, 0.30, 0.50, 1.0, and 1.5 mg/L) measured at 24, 48, 72, and 96 h post-fertilization. Values represent mean ± SD (*n* = 3 biological replicates). Asterisks indicate significant differences compared with the control at the corresponding time point (one-way ANOVA followed by Dunnett’s post hoc test, *p* < 0.05).

Hatching patterns in all BP-3 exposure groups followed a time-dependent progression (Figure 2). As expected for zebrafish embryos, hatching remained minimal at 24 h. At 48 h, embryos exposed to 0.50 and 1.50 mg/L BP-3 showed significantly lower hatching percentages compared with the control (Dunnett’s test, *p* < 0.05), indicating a transient delay in hatching. Although Fisher’s LSD test detected additional differences, these were not consistently supported by more conservative multiple-comparison analyses.

By 72 h, hatching rates increased markedly across treatments, and by 96 h, no significant differences among groups were observed. Overall, these findings suggest that BP-3 exposure may temporarily delay hatching at intermediate time points but does not produce sustained inhibition of the process. The mechanism underlying this transient effect remains to be elucidated. Although a transient delay in hatching was observed at intermediate time points, BP-3 tissue concentrations were quantified only at the 96 h endpoint. Therefore, a direct relationship between larval body burden and hatching dynamics cannot be established from the present data. Time-resolved bioaccumulation measurements would be required to clarify this potential linkage.

### 3.2. Tissue Concentrations of BP-3

**Figure 3:**
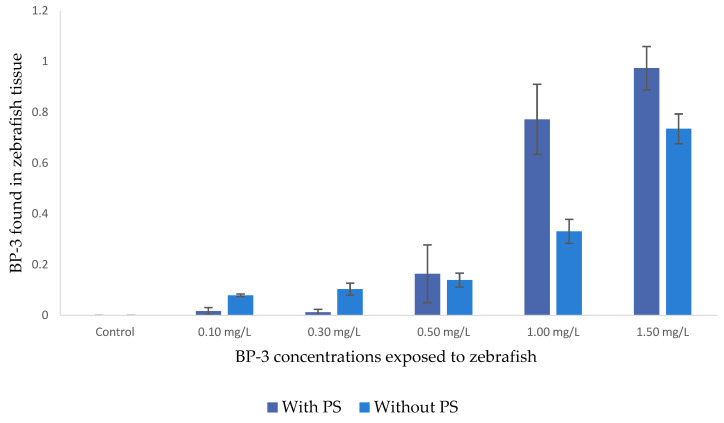
Concentrations of BP-3 detected in zebrafish larvae tissue after 96 h of exposure in the presence of PS MPs (current study) and in the absence of PS MPs (data reproduced from [8] for comparison). The prior experiment was conducted under the same exposure conditions and analytical methods. Values represent mean ± SD (*n* = 3 biological replicates). Cross-study comparisons should be interpreted with appropriate caution.

After 96 h of exposure, BP-3 concentrations in zebrafish larvae increased with nominal treatment level, demonstrating an overall dose-dependent accumulation pattern. In the present study, larvae co-exposed to PS MPs showed concentration-dependent differences in tissue BP-3 levels when compared with values previously reported for larvae exposed without MPs under equivalent experimental conditions (Figure 3) [8]. At lower exposure levels (0.10–0.30 mg/L), tissue BP-3 concentrations were lower in the presence of MPs relative to the prior single-contaminant dataset, whereas at the intermediate concentration (0.50 mg/L), differences were minimal. At higher exposure levels (1.00–1.50 mg/L), tissue concentrations were comparable or slightly higher in the presence of MPs. Because the no-MP dataset was generated in a separate experiment, these comparisons should be interpreted cautiously. Overall, the findings suggest that interactions between PS MPs and BP-3 bioaccumulation may vary with exposure concentration. These findings highlight the complexity of interactions between hydrophobic UV filters and microplastics in the early developmental stages of zebrafish [26]. However, temporal uptake dynamics were not assessed in the present study and warrant further investigation.

### 3.3. BP-3 Concentration in Exposure Water

Residual BP-3 levels in the exposure water increased with both nominal treatment concentration and exposure duration. Across all treatments, measured concentrations were consistently lower than nominal values, particularly at later time points, indicating concurrent uptake by larvae and adsorption onto PS MPs. The gradual increase in residual concentrations over time likely reflects a dynamic balance among dissolved BP-3, biological absorption, and sorption–desorption processes occurring at the MPs’ surface.

**Figure 4:**
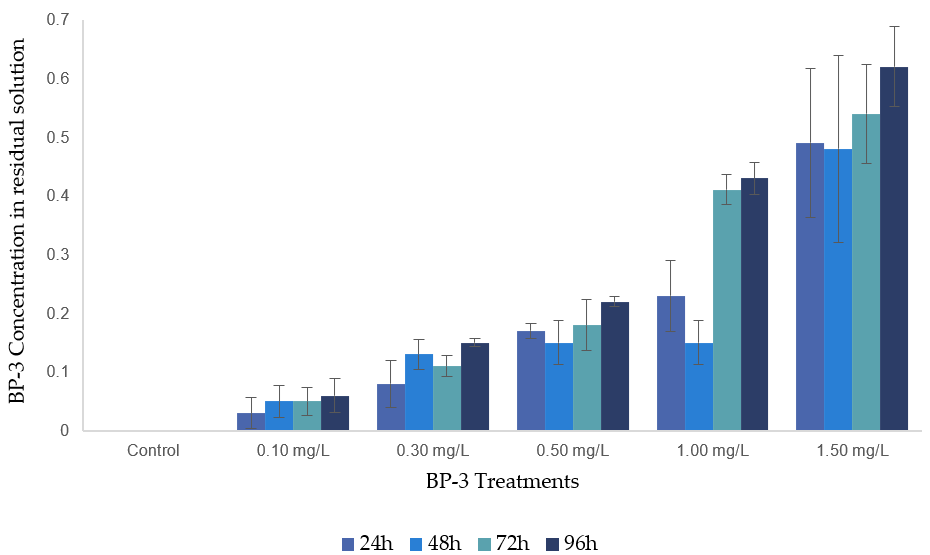
Comparison of concentrations of BP-3 found in residual treatment water for 0.00, 0.10, 0.30, 0.50, 1.0, and 1.5 mg/L of BP-3 every 24 h for 96 h. Lower concentrations in the original treatment were due to absorbance by zebrafish larvae and PS MPs *(n* = 3).

As shown in Figure 4, residual BP-3 concentrations in the exposure water exhibited clear concentration and time-dependent trends throughout the 96 h exposure period. At all nominal treatment levels, measured aqueous concentrations were consistently lower than initial values, confirming progressive removal of BP-3 from the water column over time. This reduction was most pronounced at the lowest exposure level (0.10 mg/L), where concentrations remained minimal across all sampling intervals, indicating strong partitioning of BP-3 onto PS MPs and efficient biological uptake. At intermediate exposure levels (0.30–0.50 mg/L), residual concentrations were more stable and closer to nominal values, consistent with progressive saturation of MPs’ sorption capacity [27]. These patterns indicate that adsorption and uptake processes were most effective at lower concentrations, whereas higher exposure levels exceeded the available binding capacity of MPs, leading to greater retention in the aqueous phase.

**Figure 5:**
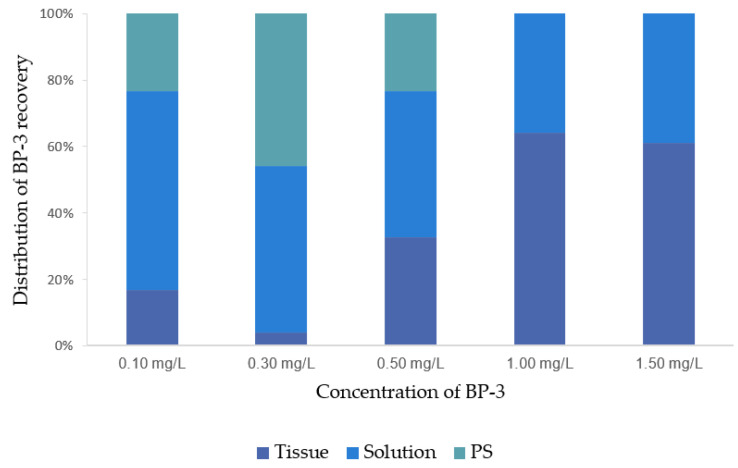
Estimated concentration of BP-3 found in PS after treatment of 0.00, 0.10, 0.30, 0.50, 1.0, and 1.5 mg/L of BP-3 for 96 h, when compared to concentrations found in zebrafish larvae tissue and residual water (*n* = 3).

Distribution analysis indicated that BP-3 partitioned between larval tissue and the remaining solution in a concentration-dependent manner (Figure 4), with an additional fraction inferred to be associated with PS MPs based on mass balance considerations (Figure 5). At the lowest exposure level (0.10 mg/L), most of the compounds remained in the dissolved phase, while a smaller fraction was recovered in larval tissue. At intermediate concentrations (0.30–0.50 mg/L), a larger proportion was associated with tissue, and the unrecovered fraction was attributed to sorption onto MPs. At the highest exposure levels (1.00–1.50 mg/L), BP-3 was primarily detected in tissue and solution. Because BP-3 associated with MPs was not directly quantified, MPs’ partitioning values represent estimated fractions derived from the difference between the nominal concentration and the measured amounts in water and tissue.

### 3.4. Adsorption of PS MPs with BP-3

To evaluate the adsorption behavior of PS MPs in the presence of BP-3, the concentration of the compound remaining in solution was quantified, and the corresponding percent recovery was determined at multiple time points over a 48 h period. In Table 1, all measured concentrations and their respective percent recoveries are presented. At the lowest initial concentration (0.10 mg/L), percent recoveries were consistently low, ranging from 1.47% to 42.13%, indicating substantial removal of BP-3 from the aqueous phase.

Hydrophobic organic compounds (HOCs), such as BP-3, exhibit a strong tendency to leave the aqueous phase and accumulate on hydrophobic surfaces, including MP particles, thereby enhancing their accumulation in water–plastic systems [25]. When contaminant concentrations are low, as typically observed in natural aquatic environments such as the sea and fresh waters, adsorption is the dominant process; however, at higher concentrations, absorption often prevails due to the larger molecular volume required for contaminant accommodation [18].

Beyond hydrophobicity, multiple interaction mechanisms mediate the association between HOCs and MPs. Including electrostatic, π-π, hydrogen bonding, van der Waals, and pore-filling interactions, with adsorption on the MP surface as the predominant process responsible for their vector properties [6,28]. The behavior previously described for the lowest initial concentration (0.10 mg/L) suggests strong sorption onto PS MPs, consistent with the interactions governing the association between MPs and organic contaminants such as BP-3.

A positive increasing trend was observed between initial concentration and percent recovery across nearly all time points, indicating that higher initial concentrations led to greater amounts of analyte remaining in solution. This trend supports the hypothesis that PS MPs possess a limited number of adsorption sites that become progressively saturated at higher analyte loads. Once these sites are occupied, the remaining compound persists in solution, resulting in higher recovery percentages [29]. This describes the initial sorption behavior of BP3 with PS MPs, where no biological compartment is present; however, at longer exposure times, the system undergoes additional redistribution processes due to slow intraparticle diffusion and reversible desorption.

Polymer composition and structure further influence sorption behavior, as glassy polymers, such as PS, exhibit internal pores that serve as strong adsorption sites, leading to slower desorption of organic contaminants [18]. PS adsorbs pollutants primarily through hydrophobic and π-π co-electron interactions, which explain its higher affinity for aromatic compounds compared to other polymers [5,12]. Additionally, environmental weathering processes, such as UV-induced photooxidation, can enhance PS sorption capacity by introducing oxygen-containing functional groups that promote hydrogen bonding with contaminants [5].

**Figure 6:**
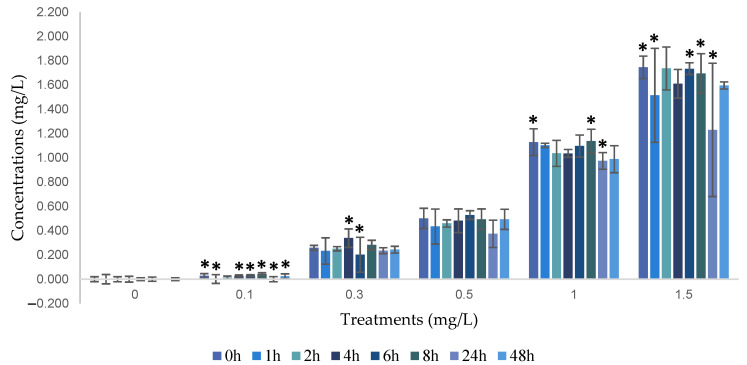
Measured concentrations of BP-3 in solution over 48 h exposure treatments at 0.00, 0.10, 0.30, 0.50, 1.00, and 1.50 mg/L (*n* = 3). Asterisks indicate significant differences among time points within the same treatment (Fisher LSD method, *p* < 0.05).

Furthermore, temporal variations were observed in Figure 6, particularly the transient decrease in measured concentrations at intermediate times followed by partial recovery, suggesting dynamic redistribution between the plastic and water phases. These trends are consistent with previous reports indicating that sorption and desorption of hydrophobic organic compounds on MPs are time-dependent processes with non-instantaneous equilibrium and partial reversibility [18]. This time-dependent sorption–desorption behavior also explains the high recovery values observed at 48 h (Table 1) across all concentrations. Although BP3 initially partitions to PS MPs, progressive desorption over time shifts the system toward an aqueous-phase-favored equilibrium, resulting in recovery percentages approaching 100% at longer exposure times.

Statistical analysis demonstrated that temporal changes in the quantified BP-3 concentrations were strongly dependent on the initial exposure concentration. At the highest concentration (1.50 mg/L), significant differences (*p* < 0.05) were observed between 24 h and several time points, including 0–24 h, 2–24 h, 6–24 h, and 8–24 h, indicating measurable temporal variation in aqueous concentrations. At 1.00 mg/L, significant differences were observed between 0 h and 24 h and between 8 h and 24 h (*p* < 0.05), suggesting gradual changes in sorption dynamics over time. In contrast, at 0.5 mg/L, no significant temporal variation was detected. At 0.3 mg/L, a significant difference between 4 h and 6 h was observed, reflecting transient fluctuations during the equilibration phase. The lowest concentration (0.10 mg/L) exhibited the greatest variability, with significant differences observed between multiple time points, including 0–24 h, 1–8 h, 4–24 h, 6–24 h, 8–24 h, and 24–48 h (*p* < 0.05), indicating unstable partitioning at low contaminant levels.

These statistical patterns are consistent with the observed recovery percentages (Table 1) and reflect the simultaneous occurrence of sorption and desorption processes. At intermediate concentrations, percent recoveries showed moderate variability, suggesting dynamic exchange between the dissolved and adsorbed fractions. The lowest recoveries were generally observed after 24 h, suggesting that BP-3 remained sequestered on PS MPs over time. In contrast, higher concentrations (1.00 and 1.50 mg/L) exhibited stable recoveries approaching or exceeding 100%, indicating the establishment of a dynamic equilibrium between the aqueous and solid phases and suggesting saturation of available adsorption sites.

This behavior is consistent with previous studies showing that adsorption onto PS MPs is strongly influenced by compound hydrophobicity, with stronger adsorption associated with lower desorption rates [29]. In particular, compounds with higher adsorption capacity tend to exhibit limited release back into the aqueous phase, supporting the sustained retention of BP-3 observed at extended exposure times.

Rapid decreases in measured concentrations were observed within the first 2 h for all treatments, particularly at concentrations ≥ 0.30 mg/L, with recoveries ranging from approximately 77% to 112%. This immediate reduction reflects rapid sorption of BP-3 onto PS MPs, consistent with BP-3′s hydrophobic character and PS’s high affinity for organic contaminants. Between 4 and 8 h, residual concentrations stabilized, with recoveries typically 90–113%, indicating that sorption equilibrium was approached during the early exposure period and that desorption was minimal.

At extended exposure times (24–48 h), concentrations remained consistently below nominal values, especially at higher treatment levels, demonstrating sustained sequestration of BP-3 by PS MPs. At 1.50 mg/L, residual concentrations ranged from approximately 1.2 to 1.6 mg/L (82–106% recovery), further supporting the persistence of adsorption and the absence of substantial release back into the aqueous phase. In contrast, the pronounced temporal variability observed at 0.10 mg/L suggests that, at low concentrations, limited adsorption site occupancy and analytical uncertainty play a greater role in influencing measured values.

Similar time-dependent behavior has been reported by Bakir et al. (2014) [25], demonstrating that sorption equilibrium may require 24–48 h to establish and that intraparticle diffusion can limit mass transfer, resulting in time-dependent redistribution and partial desorption [30]. Therefore, the concentration fluctuations observed in this study reflect a non-linear kinetic process governed by competitive adsorption, slow diffusion within the polymer matrix, and reversible partitioning, supporting the role of PS MPs as dynamic reservoirs for BP-3 in aquatic environments.

### 3.5. BP-3–Microplastic Interactions and Biological Effects

A dynamic sorption–desorption process was observed between BP-3 and PS MPs. Because marine and freshwater organisms can ingest MPs, this interaction has important implications for contaminant bioavailability and trophic transfer. Although the presence of MPs reduced the direct uptake of dissolved BP-3 by tissues, contaminated particles may still be consumed, thereby introducing an alternative exposure pathway [31]. Consequently, contaminant transfer through food webs may be altered, potentially affecting multiple life-cycle stages and propagating effects across trophic levels.

The reduction in aqueous BP-3 availability directly influenced bioaccumulation in zebrafish larvae. Larvae exposed to PS MPs accumulated significantly lower BP-3 concentrations in their tissues than those exposed in the absence of MPs. The temporal stability of reduced aqueous concentrations explains the consistently lower tissue burdens observed at 96 h, supporting the hypothesis that PS MPs effectively limit BP-3 bioavailability during exposure.

Despite reduced tissue accumulation, zebrafish embryos exhibited transient developmental effects, including delayed hatching and abnormalities in swim bladder inflation at later developmental stages. These responses suggest that early-life exposure occurred before complete sorption equilibrium was established or that even reduced BP-3 concentrations were sufficient to induce sublethal effects. Importantly, although MPs-mediated sorption reduced direct aqueous exposure and tissue accumulation, the persistence of BP-3 associated with PS particles introduced an alternative exposure pathway. Contaminated MPs may function as vectors for delayed and trophic transfer, posing potential risks to higher organisms that ingest them. Although trophic transfer was not directly evaluated in the present study, the observed association of BP-3 with PS MPs suggests a potential pathway for secondary exposure in aquatic food webs.

This interpretation is consistent with previous findings, as earlier studies have demonstrated that MPs can transfer associated contaminants across trophic levels and release them within aquatic organisms. Batel et al. showed that benzo[a]pyrene-loaded MPs were transferred from *Artemia* nauplii to zebrafish and subsequently desorbed in the intestinal tract, with fluorescence signals detected in liver tissue [27]. These findings support the hypothesis that contaminated MPs may act as vectors for trophic transfer, potentially affecting organism health and ecosystem stability. Thus, PS MPs play a dual role by decreasing short-term bioavailability while simultaneously enhancing the environmental persistence and mobility of BP-3.

### 3.6. Limitations of the Study

Several methodological considerations should be taken into account when interpreting the findings of this study. First, the MP-only sorption experiment (Section 2.7) was conducted over a 48 h period with the objective of characterizing the short-term sorption–desorption dynamics of BP-3 on PS microplastics under controlled conditions. This time window is consistent with previous studies examining the kinetics of hydrophobic organic contaminants on microplastics and was sufficient to capture the transition toward an aqueous-phase-favored equilibrium. However, longer exposure periods may reveal additional slow desorption or intraparticle diffusion processes that were beyond the scope of the present design.

Second, in the zebrafish embryo exposure experiment (Section 2.4 and Section 2.6), BP-3 quantification in larval tissue was performed only at the terminal 96 h time point. This approach followed OECD Guideline 236, which specifies continuous exposure and endpoint assessment at 96 h post-fertilization. Intermediate tissue sampling (e.g., at 24 or 48 h) was not conducted to avoid compromising embryo viability and to maintain compliance with the standardized protocol. As a result, temporal uptake dynamics, metabolic processing, and redistribution of BP-3 within larvae could not be resolved, limiting the ability to directly compare kinetic patterns between the biological system and the MP-only sorption assay.

Third, the estimated BP-3 fraction associated with PS MPs in the embryo exposure experiment was derived indirectly through mass balance calculations, as direct quantification of BP-3 on MPs was not feasible without disrupting the biological assay. These estimates should therefore be interpreted with caution, particularly at higher exposure concentrations where multiple competing processes (biological uptake, sorption, and desorption) occur simultaneously.

Finally, the study employed pristine PS microplastics, which do not fully represent environmentally aged particles. Weathering processes such as UV exposure, oxidation, and biofilm formation can alter surface chemistry and increase sorption capacity, potentially modifying BP-3 partitioning behavior in natural environments [32]. These factors should be considered when extrapolating laboratory findings to ecological scenarios.

## 4. Conclusions

This study evaluated the toxicological effects of BP-3 on early-life-stage zebrafish and its interaction with PS MPs under controlled exposure conditions. BP-3 produced measurable sublethal developmental effects, including swim bladder impairment and altered pigmentation, without a clear treatment-related increase in mortality. Tissue analysis confirmed BP-3 bioaccumulation across exposure levels.

The co-exposure experiments showed that MPs’ effect on BP-3 uptake was concentration-dependent. At lower exposure concentrations, the presence of PS MPs was associated with lower tissue BP-3 levels, whereas this mitigating effect was not consistently observed at higher concentrations, indicating a non-linear co-exposure response.

A separate time-resolved sorption experiment demonstrated rapid removal of BP-3 from the aqueous phase in the presence of PS MPs, with the greatest adsorption occurring within the first 2–4 h of exposure. Lower initial concentrations showed reduced percent recoveries, indicating stronger relative sorption. Together, higher concentrations approached equilibrium within 24–48 h, suggesting progressive saturation of adsorption sites.

These results indicate that MPs can alter the distribution of BP-3 between water and organisms. Because BP-3 associated with MPs was not directly quantified, sorption estimates were based on mass balance. Future studies should directly measure sorption capacity and evaluate ingestion-mediated and trophic transfer pathways to better define ecological risk.

## Figures and Tables

**Table 1:** Measured concentrations of BP-3 remaining in solution after exposure and their respective percent recoveries at multiple time points over a 48 h period.

Time (h)	Initial Concentration (mg/L)	Concentration Quantified (mg/L)	Recovery Percentage (%)
0	0.00	0.00 ± 0.00	0.00
0	0.10	0.02 ± 0.02	26.88
0	0.30	0.26 ± 0.02	86.15
0	0.50	0.50 ± 0.08	100.14
0	1.00	1.12 ± 0.11	112.80
0	1.50	1.74 ± 0.09	116.28
1	0.00	0.00 ± 0.00	0.00
1	0.10	0.00 ± 0.03	1.47
1	0.30	0.23 ± 0.11	77.38
1	0.50	0.43 ± 0.14	86.67
1	1.00	1.10 ± 0.02	110.22
1	1.50	1.51 ± 0.38	100.95
2	0.00	0.00 ± 0.00	0.00
2	0.10	0.01 ± 0.01	13.00
2	0.30	0.25 ± 0.02	83.48
2	0.50	0.46 ± 0.02	91.90
2	1.00	1.04 ± 0.11	103.59
2	1.50	1.74 ± 0.17	115.71
4	0.00	0.00 ± 0.00	0.00
4	0.10	0.03 ± 0.01	26.09
4	0.30	0.34 ± 0.08	112.74
4	0.50	0.48 ± 0.09	96.21
4	1.00	1.04 ± 0.03	103.52
4	1.50	1.61 ± 0.11	107.27
6	0.00	0.00 ± 0.00	0.00
6	0.10	0.03 ± 0.01	29.07
6	0.30	0.20 ± 0.14	67.17
6	0.50	0.53 ± 0.04	105.64
6	1.00	1.10 ± 0.09	109.62
6	1.50	1.73 ± 0.05	115.54
8	0.00	0.00 ± 0.00	0.00
8	0.10	0.04 ± 0.01	42.13
8	0.30	0.28 ± 0.04	93.94
8	0.50	0.49 ± 0.09	98.57
8	1.00	1.14 ± 0.10	113.68
8	1.50	1.69 ± 0.16	112.91
24	0.00	0.00 ± 0.00	0.00
24	0.10	0.01 ± 0.02	0.00
24	0.30	0.23 ± 0.02	78.25
24	0.50	0.37 ± 0.11	74.71
24	1.00	0.97 ± 0.07	97.34
24	1.50	1.22 ± 0.54	81.97
48	0.00	0.00 ± 0.00	0.00
48	0.10	0.02 ± 0.02	24.43
48	0.30	0.24 ± 0.03	80.98
48	0.50	0.49 ± 0.08	98.55
48	1.00	0.98 ± 0.11	98.81
48	1.50	1.59 ± 0.03	106.30

## Data Availability

The original contributions presented in this study are included in the article. Further inquiries can be directed to the corresponding author.

## References

[1] Peng L., Fu D., Qi H., Lan C.Q., Yu H., Ge C. (2020). Micro- and Nano-Plastics in Marine Environment: Source, Distribution and Threats—A Review. Sci. Total Environ..

[2] Fu L., Li J., Wang G., Luan Y., Dai W. (2021). Adsorption Behavior of Organic Pollutants on Microplastics. Ecotoxicol. Environ. Saf..

[3] Li Y., Li M., Li Z., Yang L., Liu X. (2019). Effects of Particle Size and Solution Chemistry on Triclosan Sorption on Polystyrene Microplastic. Chemosphere.

[4] Prajapati A., Narayan Vaidya A., Kumar A.R. (2022). Microplastic Properties and Their Interaction with Hydrophobic Organic Contaminants: A Review. Environ. Sci. Pollut. Res..

[5] Hartmann N.B., Rist S., Bodin J., Jensen L.H., Schmidt S.N., Mayer P., Meibom A., Baun A. (2017). Microplastics as Vectors for Environmental Contaminants: Exploring Sorption, Desorption, and Transfer to Biota. Integr. Environ. Assess. Manag..

[6] Koelmans A.A., Bakir A., Burton G.A., Janssen C.R. (2016). Microplastic as a Vector for Chemicals in the Aquatic Environment: Critical Review and Model-Supported Reinterpretation of Empirical Studies. Environ. Sci. Technol..

[7] Ortiz-Román M.I., Casiano-Muñiz I.M., Román-Velázquez F.R. (2024). Toxicity of UV Filter Benzophenone-3 in Brine Shrimp Nauplii (*Artemia salina*) and Zebrafish (*Danio rerio*) Embryos. J. Xenobiotics.

[8] Wang W., Ge J., Yu X. (2020). Bioavailability and Toxicity of Microplastics to Fish Species: A Review. Ecotoxicol. Environ. Saf..

[9] O’Donovan S., Mestre N.C., Abel S., Fonseca T.G., Carteny C.C., Willems T., Prinsen E., Cormier B., Keiter S.S., Bebianno M.J. (2020). Effects of the UV Filter, Oxybenzone, Adsorbed to Microplastics in the Clam *Scrobicularia plana*. Sci. Total Environ..

[10] de Sá L.C., Oliveira M., Ribeiro F., Rocha T.L., Futter M.N. (2018). Studies of the Effects of Microplastics on Aquatic Organisms: What Do We Know and Where Should We Focus Our Efforts in the Future?. Sci. Total Environ..

[11] Hüffer T., Hofmann T. (2016). Sorption of Non-Polar Organic Compounds by Micro-Sized Plastic Particles in Aqueous Solution. Environ. Pollut..

[12] Erickson R.J., McKim J.M., Lien G.J., Hoffman A.D., Batterman S.L. (2006). Uptake and Elimination of Ionizable Organic Chemicals at Fish Gills: I. Model Formulation, Parameterization, and Behavior. Environ. Toxicol. Chem..

[13] Scheele A., Sutter K., Karatum O., Danley-Thomson A.A., Redfern L.K. (2023). Environmental Impacts of the Ultraviolet Filter Oxybenzone. Sci. Total Environ..

[14] Balázs A., Krifaton C., Orosz I., Szoboszlay S., Kovács R., Csenki Z., Urbányi B., Kriszt B. (2016). Hormonal Activity, Cytotoxicity and Developmental Toxicity of UV Filters. Ecotoxicol. Environ. Saf..

[15] Rochman C.M., Manzano C., Hentschel B.T., Simonich S.L.M., Hoh E. (2013). Polystyrene Plastic: A Source and Sink for Polycyclic Aromatic Hydrocarbons in the Marine Environment. Environ. Sci. Technol..

[16] Yu F., Qin Q., Zhang X., Ma J. (2024). Characteristics and Adsorption Behavior of Typical Microplastics in Long-Term Accelerated Weathering Simulation. Environ. Sci. Process. Impacts.

[17] Rochman C.M., Hoh E., Hentschel B.T., Kaye S. (2013). Long-Term Field Measurement of Sorption of Organic Contaminants to Five Types of Plastic Pellets: Implications for Plastic Marine Debris. Environ. Sci. Technol..

[18] Zhang Y., Shah P., Wu F., Liu P., You J., Goss G. (2021). Potentiation of Lethal and Sub-Lethal Effects of Benzophenone and Oxybenzone by UV Light in Zebrafish Embryos. Aquat. Toxicol..

[19] Cormier B., Batel A., Cachot J., Bégout M.-L., Braunbeck T., Cousin X., Keiter S.H. (2019). Multi-Laboratory Hazard Assessment of Contaminated Microplastic Particles by Means of Enhanced Fish Embryo Test with the Zebrafish (*Danio rerio*). Front. Environ. Sci..

[20] Downs C.A., Kramarsky-Winter E., Segal R., Fauth J., Knutson S., Bronstein O., Ciner F.R., Jeger R., Lichtenfeld Y., Woodley C.M. (2016). Toxicopathological Effects of the Sunscreen UV Filter, Oxybenzone (Benzophenone-3), on Coral Planulae and Cultured Primary Cells and Its Environmental Contamination in Hawaii and the U.S. Virgin Islands. Arch. Environ. Contam. Toxicol..

[21] Ma W., Zeng W., Zhang D., Zhou Y., Huang Y., Hong Y. (2025). Oxidative Stress in Aquaculture: Pathogenic Mechanisms and Preventive Strategies in Farmed Aquatic Animals. Curr. Issues Mol. Biol..

[22] Batel A., Linti F., Scherer M., Erdinger L., Braunbeck T. (2016). Transfer of Benzo[*a*]Pyrene from Microplastics to *Artemia nauplii* and Further to Zebrafish via a Trophic Food Web Experiment: CYP1A Induction and Visual Tracking of Persistent Organic Pollutants. Environ. Toxicol. Chem..

[23] Kim S.W., Chae Y., Kim D., An Y.-J. (2019). Zebrafish Can Recognize Microplastics as Inedible Materials: Quantitative Evidence of Ingestion Behavior. Sci. Total Environ..

[24] Fan X., Zou Y., Geng N., Liu J., Hou J., Li D., Yang C., Li Y. (2021). Investigation on the Adsorption and Desorption Behaviors of Antibiotics by Degradable MPs with or without UV Ageing Process. J. Hazard. Mater..

[25] Bakir A., Rowland S.J., Thompson R.C. (2014). Enhanced Desorption of Persistent Organic Pollutants from Microplastics under Simulated Physiological Conditions. Environ. Pollut..

[26] Hwang J., Choi D., Han S., Jung S.Y., Choi J., Hong J. (2020). Potential Toxicity of Polystyrene Microplastic Particles. Sci. Rep..

[27] Liu J., Yuan X., Fan C., Ma G. (2024). Application of the Zebrafish Model in Human Viral Research. Virus Res..

[28] Park J.-W., Kim M., Kim S.-Y., Bae J., Kim T.-J. (2023). Biodegradation of Polystyrene by Intestinal Symbiotic Bacteria Isolated from Mealworms, the Larvae of *Tenebrio molitor*. Heliyon.

[29] Suh S., Pham C., Smith J., Mesinkovska N.A. (2020). The Banned Sunscreen Ingredients and Their Impact on Human Health: A Systematic Review. Int. J. Dermatol..

[30] He T., Tsui M.M.P., Tan C.J., Ng K.Y., Guo F.W., Wang L.H., Chen T.H., Fan T.Y., Lam P.K.S., Murphy M.B. (2019). Comparative Toxicities of Four Benzophenone Ultraviolet Filters to Two Life Stages of Two Coral Species. Sci. Total Environ..

[31] Lee W.S., Cho H.-J., Kim E., Huh Y.H., Kim H.-J., Kim B., Kang T., Lee J.-S., Jeong J. (2019). Bioaccumulation of Polystyrene Nanoplastics and Their Effect on the Toxicity of Au Ions in Zebrafish Embryos. Nanoscale.

[32] Pitt J.A., Kozal J.S., Jayasundara N., Massarsky A., Trevisan R., Geitner N., Wiesner M., Levin E.D., Di Giulio R.T. (2018). Uptake, Tissue Distribution, and Toxicity of Polystyrene Nanoparticles in Developing Zebrafish (*Danio rerio*). Aquat. Toxicol..

